# HDAC4 promotes the growth and metastasis of gastric cancer via autophagic degradation of MEKK3

**DOI:** 10.1038/s41416-022-01805-7

**Published:** 2022-05-30

**Authors:** Wei-Jie Zang, Yi-Lin Hu, Chen-Yu Qian, Ying Feng, Jia-Zhou Liu, Jun-Ling Yang, Hua Huang, Yi-Zhun Zhu, Wan-Jiang Xue

**Affiliations:** 1 Department of General Surgery, Affiliated Hospital of Nantong University, 226001 Nantong, China; 2 Research Center of Clinical Medicine, Affiliated Hospital of Nantong University, 226001 Nantong, China; 3 Medical School, Nantong University, 226001 Nantong, China; 4 Department of Clinical Biobank, Nantong University, Affiliated Hospital of Nantong University, 226001 Nantong, China; 5 Department of Pathology, Affiliated Hospital of Nantong University, 226001 Nantong, China; 6 State Key Laboratory of Quality Research in Chinese Medicine and School of Pharmacy, Macau University of Science and Technology, 999078 Macau, China

**Keywords:** Gastric cancer, Chaperone-mediated autophagy

## Abstract

**Background:**

Histone deacetylases (HDACs) have been shown to be involved in tumorigenesis, but their precise role and molecular mechanisms in gastric cancer (GC) have not yet been fully elucidated.

**Methods:**

Bioinformatics screening analysis, qRT-PCR, and immunohistochemistry (IHC) were used to identify the expression of HDAC4 in GC. In vitro and in vivo functional assays illustrated the biological function of HDAC4. RNA-seq, GSEA pathway analysis, and western blot revealed that HDAC4 activated p38 MAPK signalling. Immunofluorescence, western blot, and IHC verified the effect of HDAC4 on autophagy. ChIP and dual-luciferase reporter assays demonstrated that the transcriptional regulation mechanism of HDAC4 and ATG4B.

**Results:**

HDAC4 is upregulated in GC and correlates with poor prognosis. In vitro and in vivo assays showed that HDAC4 contributes to the malignant phenotype of GC cells. HDAC4 inhibited the MEF2A-driven transcription of ATG4B and prevented MEKK3 from p62-dependent autophagic degradation, thus activating p38 MAPK signalling. Reciprocally, the downstream transcription factor USF1 enhanced HDAC4 expression by regulating HDAC4 promoter activity, forming a positive feedback loop and continuously stimulating HDAC4 expression and p38 MAPK signalling activation.

**Conclusion:**

HDAC4 plays an oncogenic role in GC, and HDAC4-based targeted therapy would represent a novel strategy for GC treatment.

## Background

Gastric cancer (GC) is now the fifth most common malignant cancer globally, and the number of GC cases in China accounts for >40% of all new cases of GC in the world [1]. Most patients have a definite diagnosis of GC only in the advanced phase and miss the chance to undergo radical surgical treatment [2]. The prognosis of patients with GC remains poor. Therefore, it has become an urgent need to conduct in-depth research on the pathogenesis of GC and to identify effective therapeutic targets.

Mitogen-activated protein kinase (MAPK) is a class of serine/threonine protein kinases that can be activated by various intracellular and extracellular stimuli, including growth factors, hormones, oxidative stress, and endoplasmic reticulum stress [3]. The MAPK signal transduction pathway consists of three types of sequentially activated protein members: MAP kinase kinase kinase (MAPKKK or MEKK), MAP kinase kinase (MAPKK or MEK), and MAPK, which play a role in enhancing the expression of target genes or directly acting on cytoplasmic downstream kinases, regulating cell proliferation, differentiation, stress response and cell apoptosis, and other physiological activities [4]. Each MEK can be activated by at least one MEKK, and each MAPK can be activated by different MEKs, forming a complex regulatory network of MAPK [5, 6]. MAPK consists of three main subgroups: extracellular signal-regulated kinase (ERK), c-Jun amino-terminal kinase (JNK), and p38 [7, 8]. Abnormal expression or overexpression of MAPK members plays an important role in the malignant transformation and evolution of cells.

Histone deacetylases (HDACs) are a hotspot in the field of cancer drug development. Inhibition of histone deacetylation has become a recognised approach for tumour therapy [9–11]. HDACs are involved in the regulation of tumour proliferation, invasion, and migration [12, 13]. Until now, 18 HDAC subtypes have been found in the human body, which can be further subdivided into four categories: Class I HDACs (HDAC1–3 and 8) mainly exist in the nucleus, and their main function is the deacetylation of histones. Class II HDACs are further divided into Class IIA (HDAC4, 5, 7, and 9) and Class IIB (HDAC6 and 10). Class IV HDAC11 is only expressed in the brain, kidney, and testes. Class III HDAC (SIRT1–7) is associated with the yeast protein SIR2 [14, 15]. Different types of HDACs have different structures, and their effects are also different. Different subtypes of HDACs also have great differences in their baseline expression levels as well as the mechanism of action in different tumour tissues [16–18]. The role of HDACs in GC development has been studied but the mechanisms are inadequately understood [19, 20]. Therefore, it is of great significance to clarify the role and specific mechanism of action of HDACs in the progression of GC.

This study analysed the expression of HDACs in GC tissues and its influence on the prognosis of patients with GC. By using cell and animal models, the key molecular mechanisms were delineated to determine the therapeutic value of HDACs in GC.

## Materials and methods

All the experimental methods and data analysis involved in this paper are described in the Supplementary Materials, and primer sequences are shown in Table 1.

**Table 1:** Sequences of primers used for amplification of target genes.

Gene	Primer nucleotide sequence
HDAC4	Forward: 5′-CGCCTCTGTTCAACTTGTGG-3′
	Reverse: 5′-GTGAGAACTGGTGGTCCAGG-3′
HDAC2	Forward: 5′-CGTGTAATGACGGTATCATTCC-3′
	Reverse: 5′-ACCAGATAATGAGTCTGCACC-3′
MEF2A	Forward: 5′-AGCAGCCCTCAGCTCTCTTG-3′
	Reverse: 5′-GGTGAAATCGGTTCGGACTTG-3′
MEKK1	Forward: 5′-AGGTTGGCATCAAAAGGAAC-3′
	Reverse: 5′-GGCGAGATGATTGGAGTGTT-3′
MEKK2	Forward: 5′-TTTCCTCAAACGGATTT-3′
	Reverse: 5′-TGTCTTCCCATCGTCA-3′
MEKK3	Forward: 5′-AATGTGCCAACCAAGTCTCC-3′
	Reverse: 5′-TCCAGAGCACTCACCTCCTT-3′
MEKK4	Forward: 5′-CCCTCCTAACCCACACCTCATT-3′
	Reverse: 5′-CAGCACAGAGTCACCACCAGAG-3′
ATG4B	Forward: 5′-TCGGACAGCAGAACCAGC-3′
	Reverse: 5′-CCTCACCTGCGTCCATCT-3′
ATG3	Forward: 5′-ACATGGCAATGGGCTACAGG-3′
	Reverse: 5′-CTGTTTGCACCGCTTATAGCA-3′
ATG5	Forward: 5′-AAGCAACTCTGGATGGGATT-3′
	Reverse: 5′-GCAGCCACAGGACGAAAC-3′
ATG7	Forward: 5′-CAGTCCGTTGAAGTCCTC-3′
	Reverse: 5′-TCAGTGTCCTAGCCACATTAC-3′
ATG12	Forward: 5′-TGAATCAGTCCTTTGCCCCT-3′
	Reverse: 5′-CATGCCTGGGATTTGCAGT-3′
BECN1	Forward: 5′-TTTTCTGGACTGTGTGCAGC-3′
	Reverse: 5′-GCTTTTGTCCACTGCTCCTC-3′
ULK1	Forward: 5′-AGTGCAGACGGTATCATGGG-3′
	Reverse: 5′-TCTCCACCTGGGAGTGATCC-3′
GDPDH	Forward: 5′-GAGTCAACGGATTTGGTCGT-3′
	Reverse: 5′-TGGGTGGAATCATATTGGAA-3′

## Results

### HDAC4 is upregulated in GC tissues and correlated with a poor outcome

**Fig. 1:**
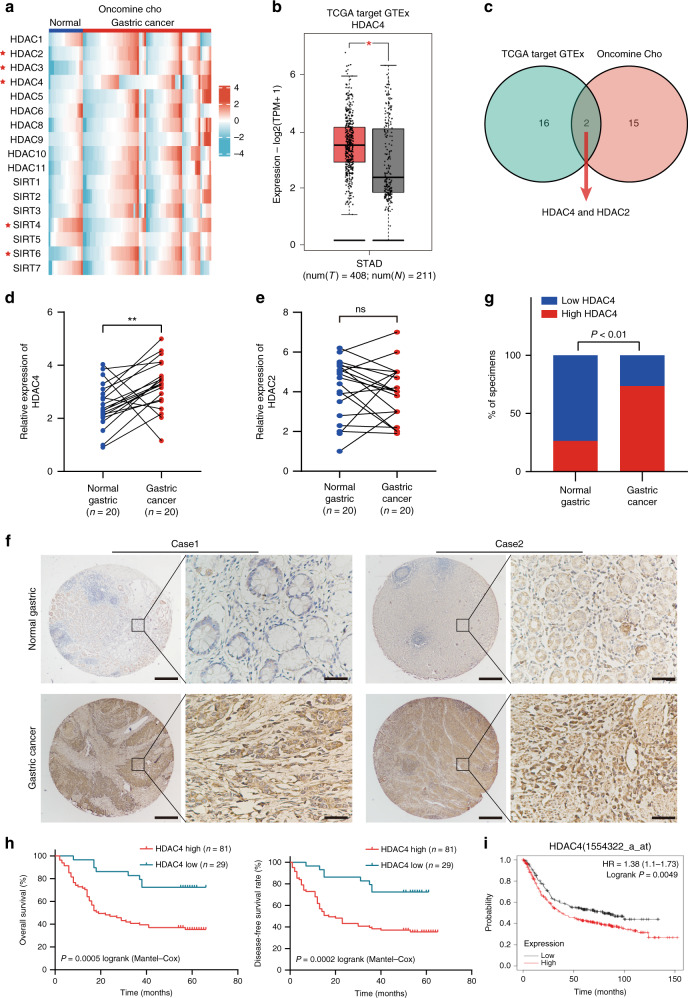
HDAC4 upregulation predicts unfavourable prognosis for patients with GC. **a** Heatmap of differentially expressed HDACs in GC based on microarray data sets (Cho). **b** The expression of HDAC4 between GC and normal tissues in TCGA target GTEx (N, normal tissue; T, tumour tissue). **c** Venn diagram displaying only HDAC2 and HDAC4 in both Cho, GSE79973, and GEPIA. **d**, **e** qRT-PCR was used to detect the expression of HDAC2 and HDAC4 in 20 matched GC samples. **f** Typical tissue microarray image analysis of HDAC4 expression in 110 patients. Scale bar, 50 μm. **g** Quantification of HDAC4 expression by immunohistochemistry analysis. **h** OS and DFS of GC patients related to HDAC4 expression by Kaplan–Meier survival curve analysis (*P* < 0.001). **i** OS of GC patients related to HDAC4 expression based on Kaplan–Meier Plotter database. *P < 0.05, **P < 0.01.

To identify HDACs associated with GC, we first screened the differentially expressed genes from 18 members of HDACs, which met the criterion of *P* value <0.05 in the Oncomine (Cho) and GEPIA databases (Fig. 1a, b and Supplementary Fig. 1A–Q). Subsequently, we selected the intersection of the above two databases and found only HDAC2 and HDAC4 to be associated with GC (Fig. 1c). Next, we detected the expression of HDAC2 and HDAC4 in 20 pairs of GC tissues by quantitative reverse transcriptase polymerase chain reaction (qRT-PCR), and the results showed that only HDAC4 is upregulated in GC tissues (Fig. 1d, e). We also verified this finding in the GSE79973 and GSE81948 data set (Supplementary Fig. 1R, S). Therefore, we chose HDAC4 for subsequent experiments. Then, we analysed HDAC4 expression by immunohistochemistry (IHC) in GC tissue microarrays with matched normal tissue samples (*n* = 110). HDAC4 expression was higher in 73.6% (81/110) of GC tissues than in normal gastric tissues (*P* < 0.01; Fig. 1f, g). Next, we analysed HDAC4 expression by IHC in GC tissue microarrays with matched normal tissue samples (*n* = 110). HDAC4 expression was higher in 73.6% (81/110) of GC tissues than in normal gastric tissues (*P* < 0.01; Fig. 1f, g). Then, we analysed the relationship between different clinicopathological aspects and HDAC4 expression level in 110 patients with GC (Table 2). HDAC4 expression was positively correlated with lymph node metastasis (*P* = 0.005), tumour, node, metastasis (TNM) stage (*P* = 0.002), and depth of invasion (*P* = 0.001). Furthermore, multiple logistic regression analysis showed that HDAC4 expression is significantly correlated with invasion depth (*P* = 0.039, odds ratio = 0.308, 95% confidence interval [CI]: 0.101–0.949). In addition, high HDAC4 expression in GC patients was significantly correlated with a shortened overall survival (OS) and disease-free survival (DFS) (Fig. 1h). Moreover, Cox multivariate analysis showed that high expression of HDAC4 is an independent predictor of OS and DFS (Table 3). The Kaplan–Meier Plotter database predicted the same result (Fig. 1I). Overall, our results showed an obvious correlation of augmented HDAC4 expression with GC progression and survival.

**Table 2:** Relationships between HDAC4 expression and clinicopathological characteristics of GC patients (*P < 0.05).

Clinicopathological parameter	HDAC4 level		Total	*P* value
	High (*n* = 81)	Low (*n* = 29)		
Gender				0.327
Male	53 (76.8%)	16 (23.2%)	69	
Female	28 (68.3%)	13 (31.7%)	41	
Age (years)				0.656
<63	43 (75.4%)	14 (24.6%)	57	
≥63	38 (71.7%)	15 (28.3%)	53	
Tumour diameter (cm)				0.069
<5	40 (66.7%)	20 (33.3%)	60	
≥5	41 (82.0%)	9 (18.0%)	50	
Tumour location				0.307
Up	15 (83.3%)	3 (16.7%)	18	
Down/middle	66 (71.7%)	26 (28.3%)	92	
Tumour differentiation				0.881
Poor	32 (74.4%)	11 (25.6%)	43	
Well/moderate	49 (73.1%)	18 (26.9%)	67	
CEA level (ng/ml)				0.638
≤2	35 (71.4%)	14 (28.6%)	49	
>2	46 (75.4%)	15 (24.6%)	61	
TNM stage				0.002*
I/II	31 (59.6%)	21 (40.4%)	52	
III/IV	50 (86.2%)	8 (13.8%)	58	
Depth of invasion				0.001*
T1 + T2	22 (55.0%)	18 (45.0%)	40	
T3 + T4	59 (84.3%)	11 (15.7%)	70	
Lymph node metastasis				0.005*
Negative	37 (62.7%)	22 (37.3%)	59	
Positive	44 (86.3%)	7 (13.7%)	51

**Table 3:** Univariate and multivariable analyses of OS and DFS in GC patients (*P < 0.05).

Variable	OS			DFS		
	Univariate analysis	Multivariable analysis		Univariate analysis	Multivariable analysis	
	*P* > |*z*|	*P* > |*z*|	HR (95% CI)	*P* > |*z*|	*P* > |*z*|	HR (95% CI)
HDAC4 expression						
Low (*n* = 28) vs. high (*n* = 82)	0.001*	0.037*	2.419 (1.057–5.536)	0.001*	0.033*	2.473 (1.075–5.694)
Gender						
Male (*n* = 69) vs. female (*n* = 41)	0.107			0.130		
Age (years)						
<63 (*n* = 57) vs. ≥54 (*n* = 53)	0.244			0.270		
Tumour differentiation						
Poor (*n* = 43) vs. well/moderate (*n* = 67)	0.016*			0.017*		
Tumour diameter (cm)						
<5 (*n* = 60) vs. ≥5 (*n* = 50)	0.090			0.106		
CEA level (ng/ml)						
≤2 (*n* = 49) vs. >2 (*n* = 61)	0.524			0.511		
Depth of invasion						
T1 + T2 (*n* = 40) vs. T3 + T4 (*n* = 70)	<0.001*	0.002*	4.024 (1.663–9.734)	<0.001*	0.001*	4.265 (1.758–10.345)
Lymph node metastasis						
Negative (*n* = 59) vs. positive (*n* = 51)	<0.001*	<0.001*	7.527 (3.609–15.698)	<0.001*	<0.001*	8.074 (3.842–16.965)
Tumour location						
Up (*n* = 18) vs. down/middle (*n* = 92)	0.879			0.858		

### HDAC4 promotes GC cell proliferation, migration, and invasion in vitro and in vivo

The expression of HDAC4 was found to be increased in GC cell lines compared with the GES-1 cell line by qRT-PCR and western blotting (Supplementary Fig. 2A, B). To explore whether HDAC4 regulated the biological behaviours of GC cells, we stably transfected HDAC4 with three HDAC4 knockdown lentiviruses (Sh-HDAC4, Sh-HDAC4#2, and Sh-HDAC4#3) in SGC7901 and BGC823 cells. The efficiency was measured by western blotting (Supplementary Fig. 2C). HDAC4 knockdown in both SGC7901 and BGC823 cells significantly suppressed cell proliferation, migration, and invasion in vitro (Supplementary Fig. 2D–I). Therefore, the Sh-HDAC4 lentivirus with the highest knockdown efficiency was selected for subsequent experiments.

**Fig. 2:**
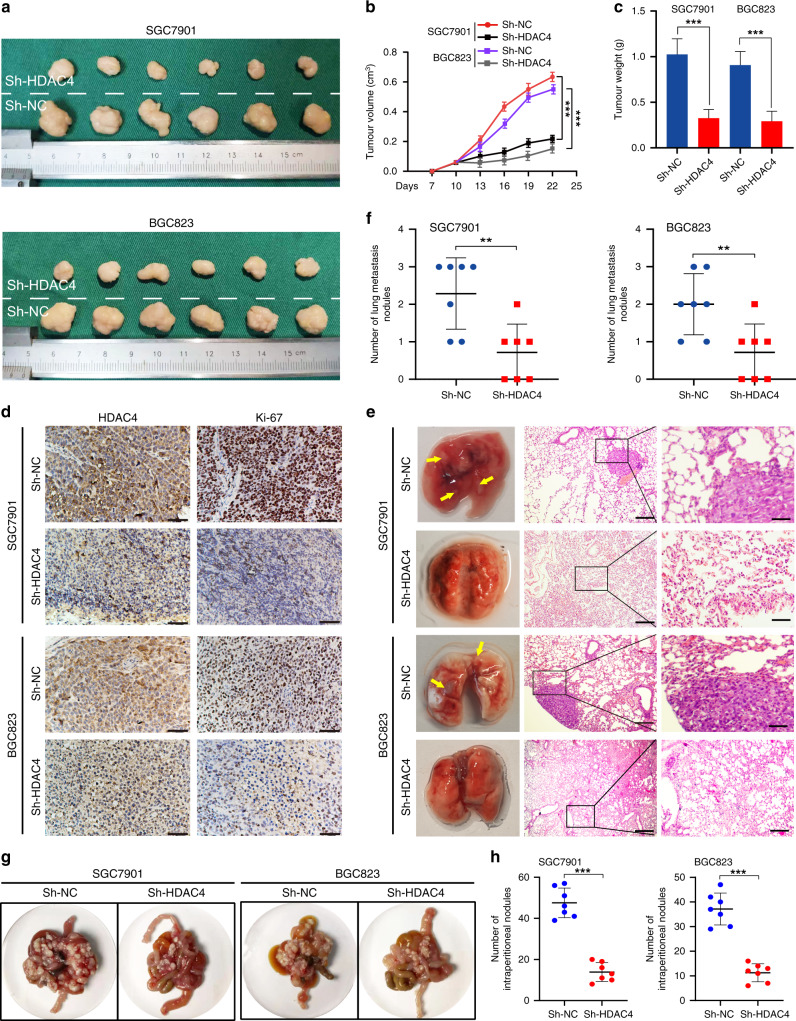
Effect of HDAC4 on tumorigenicity, lung metastasis, and abdominal metastasis in vivo. **a** Typical images of subcutaneous xenograft tumour. **b**, **c** Xenograft tumour growth and weight (*n* = 6). **d** IHC staining intensity of Ki-67 in different groups. Scale bar, 50 μm. **e** Typical images of pulmonary metastasis. Left: representative metastatic nodules of the lung; the lesions are marked with arrows. Right: HE staining of typical pulmonary nodules (scale bar, 25 μm). **f**, **h** Number of metastatic nodules. Data are the mean ± SD (*n* = 7). **g** Representative images and numbers of intraperitoneal metastatic nodules in nude mice injected with recombinant GC cells and control cells. **P < 0.01, ***P < 0.001.

To further verify the role of HDAC4 in GC cell proliferation and metastasis in vivo, we established a xenograft tumour model. The tumour growth was slower and tumour volume and weight were lower in the Sh-HDAC4 group than in the control group (Fig. 2a–c). In addition, IHC analysis revealed decreased expression of Ki-67 in the subcutaneous tumours in HDAC4 knockdown GC cells (Fig. 2d). In the mouse model of lung and abdominal metastasis, we observed that the number of metastatic tumour nodules is lower in the Sh-HDAC4 group than in the control group (Fig. 2e–h). Together, these results demonstrated that HDAC4 plays a pro-oncogenic role in GC by promoting tumour cell growth and migration.

### HDAC4 promotes malignant biological behaviours of GC cells via the p38 MAPK signalling pathway

**Fig. 3:**
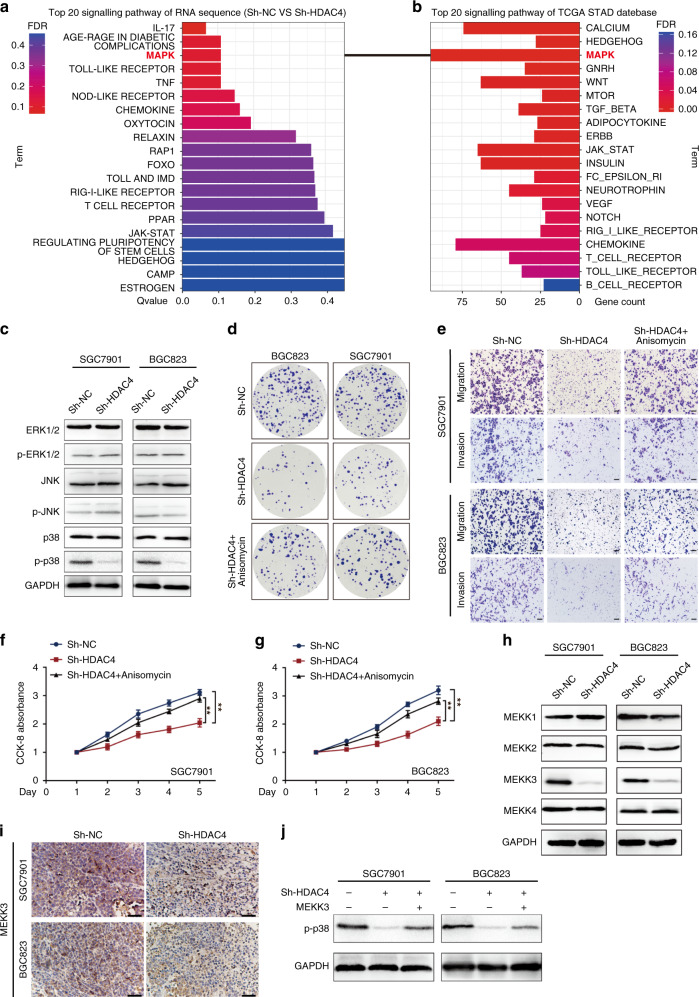
HDAC4 promotes the progression of GC via the p38 MAPK signalling pathway. **a** Top 20 signalling pathway maps enriched in the RNA Sequence and **b** TCGA STAD databases by GSEA. **c** Western blot analysis was used to detect the effect of HDAC4 knockdown on the expression of MAPK-related signalling components in GC cells; GAPDH is used as a loading control. **d**, **f**, **g** Anisomycin reverses the inhibitory effect of HDAC4 knockdown on the proliferation of GC cells as shown by the colony formation and CCK-8 assays. **e** Anisomycin reverses the inhibitory effect of HDAC4 knockdown on GC cell migration and invasion as shown in Matrigel Transwell assays of GC cells. **h** The expression of MEKK1/2/3/4 was detected by western blotting in GC cells. **i** Immunohistochemistry analysis of MEKK3 in subcutaneous tumour tissue. Scale bar, 50 μm. **j** In Sh-HDAC4 cells, MEKK3 was overexpressed to detect p-p38 expression level. **P < 0.01.

To uncover the molecular mechanism by which HDAC4 regulates GC tumour progression, we compared the transcriptome profiles of SGC7901 cells with stable HDAC4 knockdown with controls by RNA-seq analysis. Differentially expressed genes were mainly enriched in the top 20 signalling pathways according to the *Q*-value (Fig. 3a) and the top 20 signalling pathways with statistical significance in geneset enrichment analysis (GSEA) pathway analysis according to the TCGA STAD database (Fig. 3b). The MAPK signalling pathway was the only signalling pathway in the top 20 transcriptome sequencing and GSEA pathway analysis of TCGA STAD (Supplementary Fig. 3A). We further investigated which of the three subgroups of MAPK signalling is responsible for the carcinogenic function of HDAC. We found that p-p38 is remarkably downregulated in HDAC4-knockdown cells compared to control cells (Fig. 3c). To understand the importance of the p38 MAPK pathway in HDAC4-mediated GC, we performed rescue experiments using the p38 MAPK activator anisomycin (Supplementary Fig. 3B). Anisomycin restored the tumour suppression caused by HDAC4 knockdown (Fig. 3d–g and Supplementary Fig. 3C–F), suggesting that HDAC4 acts as an upstream effector of p38.

We next examined which major upstream components of the p38 MAPK signalling pathway might be regulated by HDAC4. When p38 MAPK signalling is activated, the signal transduction first occurs via MEKK. Therefore, we investigated whether HDAC4 was upstream of MEKK. Moreover, it is well known that the acetylation reaction catalysed by HDAC4 plays an important role in the transcriptional regulation of MEEK2 [21]. Therefore, we first detected the mRNA expression of MEKK1, 2, 3, and 4 (Supplementary Fig. 3G, H) but found no change in the expression after HDAC4 knockdown. Unexpectedly, we found decreased protein expression of MEKK3 in HDAC4 knockdown cells rather than MEKK1, 2, and 4 (Fig. 3h). IHC staining further confirmed that HDAC4 knockdown inhibits MEKK3 expression in the subcutaneous GC xenograft tumour model (Fig. 3i). In addition, MEKK3 overexpression reversed the effect of HDAC4 knockdown on p38 signalling (Fig. 3j). Taken together, these results showed that HDAC4 knockdown attenuates p38 MAPK signalling by promoting the protein expression of MEKK3, which is involved in the suppression of oncogenic properties in GC.

### HDAC4 knockdown-induced autophagy contributes to the degradation of MEKK3 in a p62-dependent manner

**Fig. 4:**
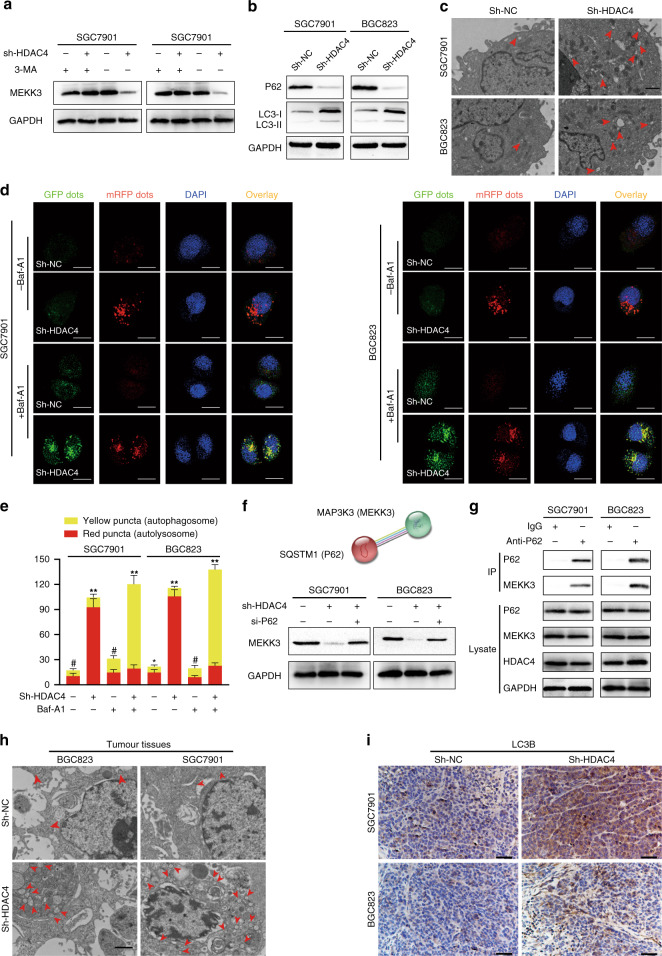
HDAC4 knockdown induces autophagic degradation of MEKK3 in GC cells and tumour tissues. **a** Western blot revealed that the autophagy inhibitor 3-MA inhibits the degradation of MEKK3 protein. **b** The STRING database was used to predict the protein interactions between P62 and MEKK3 (green, blue, purple, and black lines indicate, respectively, gene neighbourhood, the from curated databases; experimentally determined and co-expression). **c** Co-immunoprecipitation was used to detect the binding relationship between P62 and MEKK3 in GC cells. Anti-P62 antibodies were used for the co-immunoprecipitation of MEKK3 and P62, then western blot analysis was used to detect the protein levels of P62 and MEKK3. **d** Western blot showed that si-p62 can rescue MEKK3 protein level in HDAC4 knockdown GC cells. **e** The protein levels of LC3II and p62 were detected in the HDAC4 knockdown cells by western blot analysis. **f** Typical transmission electron microscopic images of endogenic autophagic double-membrane structures in HDAC4 knockdown and control cells. The autophagosomes or autolysosomes are indicated by red arrows. Scale bar, 1 μm. **g** Both control and HDAC4 knockdown cells were transfected with lentivirus containing mRFP-GFP-LC3. **h** Quantification of LC3 dots (red dots for autophagosomes and yellow dots for autolysosomes). Scale bar, 20 μm. **i** Xenografted tumours were subjected to transmission electron microscopy; red arrows indicate autophagosomes or autolysosomes. **j** Immunohistochemistry analysis of LC3B in tumours. Scale bar, 50 μm. **P < 0.01, ^#^not significant.

To identify the protein degradation pathways that might be regulated by MEKK3 stability upon HDAC4 knockdown, we first examined the protein stability of MEKK3 in HDAC4 knockdown cells in the presence of the proteasome inhibitor MG132, the autophagy inhibitor 3-methyladenine (3-MA), or dimethyl sulfoxide as control. We found that 3-MA restores the levels of MEKK3 protein but not MG132 (Fig. 4a and Supplementary Fig. 4A, B). Therefore, we hypothesised that HDAC4 mediates MEKK3 degradation through the autolysosome pathway. Autophagy can degrade cargos using autophagy cargo receptor proteins that bind to and bring substrates to the autophagosome for degradation. p62 is a well-known autophagy cargo receptor that interacts with substrates for their degradation. To identify whether p62 was involved in MEKK3 degradation in GC cells, we used the STRING database and found an interaction between MEKK3 and p62 (Fig. 4b). Co-immunoprecipitation assay further confirmed a direct interaction between p62 and MEKK3 in GC cells (Fig. 4c). Importantly, si-p62 could rescue MEKK3 protein level in HDAC4 knockdown GC cells (Fig. 4d). These results indicated that p62 is critical for the HDAC4-mediated autophagic degradation of MEKK3.

Next, to examine whether HDAC4 could inhibit autophagy in GC cells to prevent MEKK3 from p62-mediated autophagic degradation, we measured the protein expression levels of autophagy-related markers. We found that HDAC4 knockdown potently increases LC3-II protein level but downregulates p62 protein level (Fig. 4e). In addition, transmission electron microscopy (TEM) revealed that HDAC4 knockdown cells contain abundant autophagic vacuoles (Fig. 4f). After GFP-mRFP-LC3 lentivirus transfection, the numbers of red puncta (autolysosomes) were increased in HDAC4-knockdown GC cells, which indicated an increased autophagic flux. More yellow dots (autophagosomes) were observed when the autophagic flux was blocked by bafilomycin A1 (autophagosome/lysosomal fusion inhibitor) (Fig. 4g, h). In addition, we studied the effect of HDAC4 knockdown on GC autophagy in vivo. TEM revealed that HDAC4 knockdown significantly increases the number of autophagosomes (double-membrane structures) (Fig. 4i). IHC staining of LC3B was increased in xenografts of HDAC4-knockdown cells (Fig. 4j). Together, the results suggest that HDAC4 knockdown suppresses the level of autophagy in GC cells to increase MEKK3 degradation via p62-dependent selective autophagic degradation.

### ATG4B activity is essential for HDAC4-induced autophagic MEKK3 degradation

**Fig. 5:**
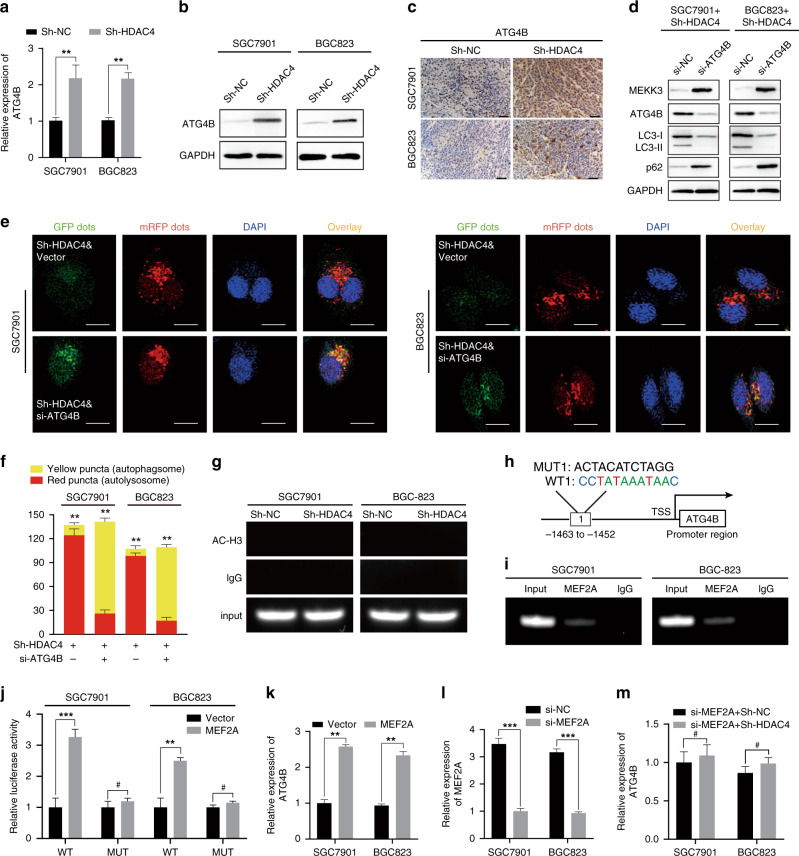
ATG4B activity promotes the HDAC4-knockdown-induced autophagic degradation of MEKK3. **a**, **b** The level of ATG4B in HDAC4-knockdown cells was detected using qRT-PCR and western blot. **c** IHC also showed the same results as protein levels of ATG4B. Scale bar, 50 μm. **d** Protein levels of LC3-II, p62, MEKK3, and ATG4B were detected by western blot analysis. **e**, **f** Detection of autophagic flux by the GFP-mRFP-LC3 lentivirus in Sh-HDAC4 GC cells with or without si-ATG4B. Scale bar, 20 μm. **g** ChIP analysis showing histone H3 acetylation (AC) at ATG4B on the promoter. **h** Binding sites of MEF2A in the promoter region of ATG4B according to JAPAR database. **i** The direct binding relationship between MEF2A and ATG4B was demonstrated by ChIP assay. **j** Luciferase reporter assay of ATG4B-WT/Mut after transfection of the MEF2A plasmid. **k** qRT-PCR analysis of ATG4B in GC cells after MEF2A overexpression. **l** qRT-PCR analysis revealed that the level of MEF2A in GC cells is suppressed. **m** qRT-PCR analysis of the relative expression of ATG4B in si-MEF2A cells with or without HDAC4 knockdown. *P < 0.05, **P < 0.01, ***P < 0.01, ^#^not significant.

To detect whether the underlying mechanism of HDAC4-induced GC cell autophagy was attributed to its ability to repress the transcription of key genes that regulated autophagy, we further analysed the differentially expressed autophagy-related genes in the transcriptome sequencing and found that the expression of only ATG4B is significantly increased in HDAC4-knockdown cells, which was verified by qRT-PCR (Fig. 5a and Supplementary Fig. 4C). ATG4B serves as a priming and delipidation enzyme whose fine regulation is essential for autophagy [22, 23]. To verify the reliability of the transcriptome sequencing, we examined the effect of HDAC4 on the expression of ATG4B. Western blot analysis showed that HDAC4 knockdown increases the expression of ATG4B in SGC7901 and BGC823 cells (Fig. 5b). Notably, IHC showed that ATG4B expression is upregulated in the xenografts of HDAC4-knockdown cells (Fig. 5c). When ATG4B was knocked down, the expression of autophagy-related markers was reduced and MEKK3 degradation induced by HDAC4 knockdown was blocked (Fig. 5d). The silencing of ATG4B also completely reversed the autophagic flux induced by HDAC4 knockdown (Fig. 5e, f).

Histone acetylation is one of the key mechanisms of gene transcriptional regulation [24], but we found that, after knocking down HDAC4, the histone H3 acetylation level of the ATG4B promoter did not change (Fig. 5g). HDAC4 binds to the transcription factor MEF2A, thereby inhibiting its transcriptional regulating ability [21, 25, 26]. We sought to determine whether HDAC4 repressed ATG4B expression via MEF2A. Then, we found that MEF2A has only one potential binding site in the ATG4B promoter region based on the JASPAR database (Fig. 5h). To further study the direct regulation of ATG4B by MEF2A combined with the promoter of ATG4B, we conducted a chromatin immunoprecipitation (ChIP) assay (Fig. 5i). Luciferase reporter assays suggested that MEF2A activates ATG4B transcription, and overexpression of MEF2A can improve ATG4B expression in SGC7901 and BGC823 cells (Fig. 5j, k). After silencing MEF2A expression, HDAC4 knockdown had little effect on ATG4B expression in GC cells (Fig. 5l, m). The above results suggest that knockdown of HDAC4 expression induces GC cell autophagy by reducing the transcriptional activation of MEF2A on ATG4B.

### HDAC4/ATG4B/p38/USF1 forms a positive feedback loop

**Fig. 6:**
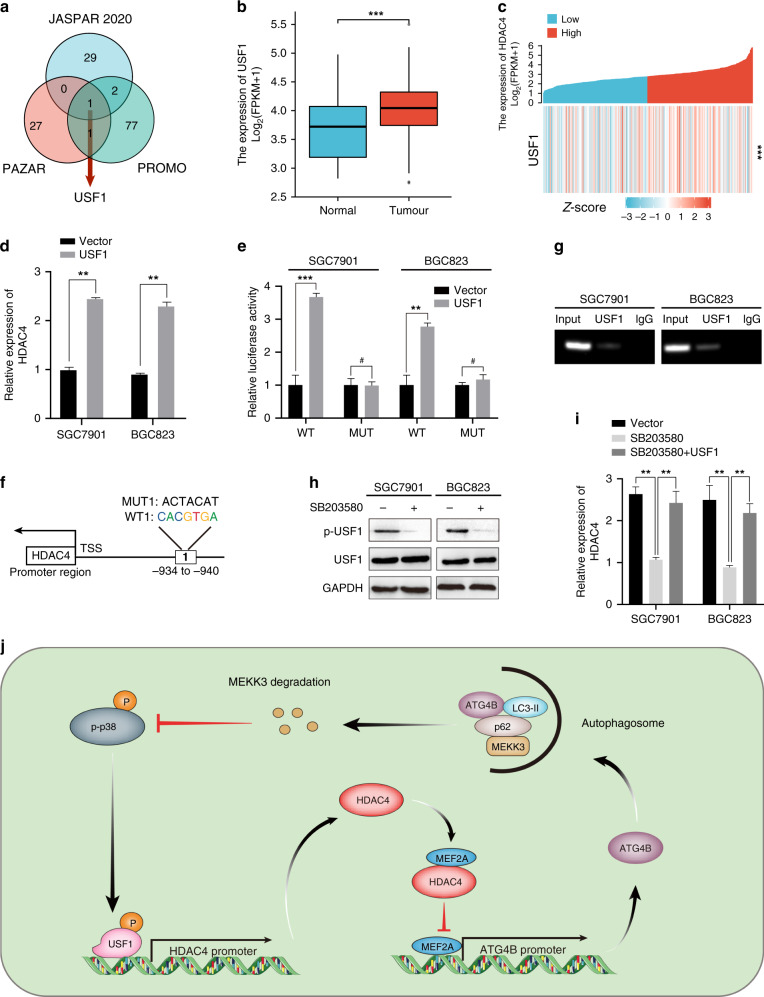
HDAC4/ATG4B/MEKK3/p38 forms a positive feedback loop. **a** Cross-prediction from three online databases (JASPAR, PROMO, and PAZAR). **b** The expression of USF1 was upregulated in the TCGA STAD database. **c** USF1 expression was correlated with HDAC4 expression based on analysis of the TCGA STAD database. **d** qRT-PCR analysis of HDAC4 in GC cells after USF1 overexpression. **e** Luciferase reporter assay of HDAC4 WT and Mut after transfection of the USF1 plasmid. **f** The potential binding sites of USF1 in the HDAC4 promoter region based on analysis of the online database. **g** The direct binding relationship between USF1 and HDAC4 was demonstrated by ChIP assay. **h** Western blot detected the protein level of USF1 and p-USF1 after application of SB203580. **i** qRT-PCR analysis of HDAC4 expression in GC cells with USF1 overexpression with or without SB203580. **j** Schematic representation of the positive feedback loop. **P < 0.01, ***P < 0.001, ^#^not significant.

To investigate the molecular mechanism by which HDAC4 is upregulated in GC, we first detected whether there was any potential transcription factor that could bind to the promoter of HDAC4 to elevate its expression. According to the intersection of candidate transcription factors for HDAC4 in PROMO, PAZAR, and JASPAR databases, Upstream Stimulatory Factor 1 (USF1) was identified as the only transcription factor that could bind to the HDAC4 promoter in all three databases (Fig. 6a). By analysing the TCGA STAD data set, we found that the expression of USF1 in GC is significantly upregulated (Fig. 6b) and is positively correlated with the expression of HDAC4 (*R* = 0.180, *P* < 0.001; Fig. 6c). The ectopic expression of USF1 could increase HDAC4 mRNA expression in GC cells (Fig. 6d). Dual-luciferase reporter assays showed that overexpression of USF1 enhances the luciferase activity driven by the HDAC4 promoter. However, when the potential binding site of USF1 in the HDAC4 promoter region was mutated, overexpression of USF1 could not affect the luciferase activity driven by mutated HDAC4 promoter (Fig. 6e, f). ChIP assays further demonstrated that USF1 could bind the HDAC4 promoter (Fig. 6g). These results indicated that USF1 interacts with the HDAC4 promoter and elevates HDAC4 mRNA expression in GC cells. Interestingly, it has been reported that USF1 is a downstream target of p38 MAPK signalling [27]. Western blot confirmed that SB203580 could inhibit the phosphorylation level of USF1 and HDAC4 mRNA expression in GC cells, whereas the upregulation of USF1 partially reverses this phenomenon (Fig. 6h, i). We then revealed a positive regulatory feedback loop formed by the HDAC4/ATG4B/MEKK3/p38 axis, which results in the continuous stimulation of HDAC4 expression and the sustainable activation of p38 MAPK signalling.

## Discussion

HDAC4 belongs to class IIA HDAC and is located on chromosome 2q37.2. It is highly expressed in the brain, heart, and skeletal muscle, as well as in various malignant tumours, including oesophageal cancer, glioblastoma, multiple myeloma, epithelial cancer, and colon cancer [28]. Its expression is associated with poor prognosis and drug resistance to chemotherapy. Spaety et al. found through biochemistry analysis that the high expression of HDAC4 in GC tissues is related to the molecular typing and poor prognosis of GC [29]. We demonstrate the same results using real clinical data. Kang et al. found that HDAC4 promotes GC progression via p21 repression [30]. We found a different mechanism: HDAC4 facilitates the progression of GC mainly by activating the p38 MAPK pathway. Our results suggest that high expression of HDAC4 may be a poor predictor of GC.

As an important pathway of intracellular protein degradation, the autophagy-lysosomal system plays an important role in both nutrient cycling and scavenging and maintenance of stability [31]. Target proteins degraded by the autophagy-lysosomal system, such as WNT and Keap1, first bind to the key autophagy protein p62/LC3B and are recognised by the receptor proteins [32, 33]. Then they are wrapped by the autophagosome with a bilayer membrane structure, after which they enter the autophagy lysosomes to complete the autophagic degradation of the proteins. We found that HDAC4 knockdown enhances the autophagic degradation of MEKK3 and reduces the expression of MEKK3 in cells, thus inhibiting the activation of the MAPK pathway and the proliferation, migration, and invasion of GC cells.

HDAC4 plays different roles by regulating autophagy. After HDAC4 interacts with autophagy-related microtubule-associated protein 1S (MAP1s), the acetylation level of MAP1s decreases, and it becomes unstable. This inhibits autophagy and promotes the accumulation of MHTT aggregates, causing the occurrence of Huntington’s disease. The polyamine spermidine can improve MAP1s instability induced by HDAC4 and inhibit the occurrence of cirrhosis and hepatocellular carcinoma by promoting autophagy [34]. In diabetic nephropathy, HDAC4 promotes the deacetylation of signal transduction and transcriptional activator 1 (STAT1), and activated STAT1 inhibits podocyte autophagy, thereby inducing podocyte injury [35, 36]. However, during vascular inflammation, the increased expression of HDAC4 can reduce the acetylation of FoxO3a in vascular endothelial cells, and activated FoxO3a can promote the transcription of autophagy-related genes ATG5 and LC3B, thereby inducing the autophagy of vascular endothelial cells [37]. In our study, HDAC4 inhibited the transcription of the autophagy-related gene ATG4B and consequently autophagy in GC.

Traditional HDACs contain the amino acid tyrosine in their enzyme active region; however, for type II HDACs, the tyrosine is replaced by histidine, so that their activity is >1000 times lower than that of type I HDACs. Class II HDACs have a type of protein structure that has a specific amino acid sequence targeting the acetyl modification of lysine and can recruit HDAC3. HDAC3 can perform the deacetylase activity in case of class II HDAC deletion and can continue to bind to the NCoR/SMRT transcription co-inhibitory complex, remove the acetyl groups of histones and non-histone proteins, and inhibit DNA transcription [38]. Non-histone proteins studied in recent years mainly include runt-associated transcription factor 2, hypoxic-inducible factor-1α, and STAT1 [39–41]. Class II HDACs can also bind to transcription factors such as MEF2s, thereby inhibiting the transcription of genes regulated by these transcription factors [42]. Our study also confirmed that HDAC4 in GC cells inhibits the expression level of ATG4B by inhibiting the effect of MEF2A on the transcription of ATG4B, thus inhibiting the autophagy of GC cells.

In conclusion, our study confirmed that HDAC4 plays an important role in the development of GC, and high HDAC4 expression can be used as an independent predictor of poor prognosis of GC. High expression of HDAC4 inhibits the transcriptional activity of MEF2A, which in turn inhibits the transcription of ATG4B, thereby inhibiting the autophagy of GC cells, reducing the degradation of MEKK3, activating p38, and promoting the growth and metastasis of GC. Therefore, HDAC4 can be used as a new potential GC therapeutic target.

## Supplementary information

Supplementary material and figure legends

Supplementary figure 1.tif

Supplementary figure 2.tif

Supplementary figure 3.tif

Supplementary figure 4.tif

checklist

## Data Availability

The data that support the findings of this study are available on request from the corresponding author.
