# Supplementary material for: HDAC4 promotes the growth and metastasis of gastric cancer via autophagic degradation of MEKK3
Source: Br J Cancer. 2022 May 30;127(2):237–48. doi: 10.1038/s41416-022-01805-7 (PMC9296555; doi:10.1038/s41416-022-01805-7)
Supplement: Supplementary file 1 — Supplementary material and figure legends [file 41416_2022_1805_MOESM1_ESM.docx]

**2. Materials and Methods**

2.1 Clinical samples

GC tissue specimens (n=110) and matched normal tissues which were enrolled at Affiliated Hospital of Nantong University (Nantong, China) from 2010 to 2011 in department of general surgery. Detailed clinical data of GC patients are shown in Table 1. Follow-up of the patients ended in August 2015 (median follow-up 37 months; range, 2-66 months). Another cohort of 20 fresh GC tissues and adjacent normal tissues were collected between 2019 and 2020 for qRT-PCR assay from the same source. All patients with GC were pathologically confirmed by two independent pathologists and had not received any adjuvant radiotherapy, chemotherapy, or immunotherapy prior to radical surgery. This study was approved by the Human Research Ethics Committee of the Affiliated Hospital of Nantong University, and each patient provided written informed consent.

**2.2 Cell culture and reagents**

A gastric mucosa cell line (GES-1) and seven GC cell lines (MGC803, MKN45, MKN1, BGC823, SGC7901, AGS, and HGC27) were purchased from GeneChem (Shanghai, China). GES-1 and GC cells were cultured in RPMI-1640 medium (ScienCell Research Laboratories, Shanghai, China) and supplemented with 10% fetal bovine serum (Clark, Shanghai, China) and 100 U/mL penicillin–streptomycin (Life Technologies, Shanghai, China). Anisomycin, MG132 and SB203580 were purchased from Beyotime (Shanghai, China), bafilomycin A1 and DAPI were purchased from Life Technologies (Shanghai, China), and 3-methyladenine (3-MA) were purchased from Solarbio (Beijing, China).

**2.3 Plasmids, siRNAs, Lentiviral vectors and cell transfections**

Full-length cDNAs of human ATG4B, MEF2A and USF1 were cloned into GV141 vector (GeneChem). The wild type ATG4B promoter (WT-ATG4B) and a promoter with mutated MEF2A-binding sites (Mut-ATG4B) were cloned into GV248 luciferase plasmid vector (GeneChem). The wild type HDAC4 promoter (WT-HDAC4) and a promoter with mutated USF1-binding sites (Mut-HDAC4) were cloned into GV248 luciferase plasmid vector (GeneChem). The short-hairpin RNA (shRNA) lentiviral targeting the HDAC4 (Sh-HDAC4, Sh-HDAC4#2 and Sh-HDAC4#3) was synthesized by GeneChem and the targeting sequence as followed: Sh-HDAC4: TTCTCCGAACGTGTCACGT; Sh-HDAC4#2: AGGCAGCGCCAGUACUCA and Sh-HDAC4#3: GCUAUGACGAUGGGAACUUTT. The negative control shRNA consisted of an no homology sequence to any known human gene (Sh-NC). siRNA sequences corresponding to si-p62, si-ATG4B and si-MEF2A were synthesized by Genepharma (Suzhou, China) The target sequences were as follows: GGACCCAUCUGUCUUCAAATT (si-p62), CAGTATCCAAACGGGCTCTG (si-ATG4B), GGGCAGUUAUCUCAGGGUUTT (si-MEF2A). The negative control (NC) was provided by Genepharma. The plasmids and abovementioned oligonucleotides GC cells were transfected into GC cells by using Lipofectamine 3000 (Invitrogen, Carlsbad, California, USA) following the manufacturer’s instructions. For lentiviral transfection were performed as described previously(1). Neomycin (G418; Roche, Indianapolis, IN, USA) was used to screen stably transfected GC cells for more than two weeks.

**2.4 RNA isolation and quantitative real-time PCR (qRT-PCR)**

RNA isolation and qRT-PCR were performed previously(2). Primer sequences are listed in Table S3. GAPDH mRNA was used as a loading control. All primers were synthesized by Sangon Biotech (Shanghai, China).

**2.5 Western blot**

Protein extraction and western blotting were consistent with this paper(3). The following primary antibodies were used in our western blotting analysis: anti-JNK (51151-1-AP), anti-P62 (18420-1-AP), anti-p‐ERK1/2 (28733-1-AP), anti-ERK1/2 (16443–1-AP), anti-HDAC4 (17449-1-AP), anti-GAPDH(60004-1-Ig), anti-MEKK3 (21072-1-AP), anti-MEKK1(19970-1-AP), anti-MEKK2 (55106-1-AP), anti-MEKK4 (21610-1-AP), anti-p38 (14064-1-AP), anti-ATG4B (67727-1-Ig), anti-USF1 (22327-1-AP) and anti-IgG (A21020-1) from Proteintech (Wuhan, China); anti-IgG (#14705) from Cell Signaling Technology (Boston, USA), anti-p‐p38 (D‐8, sc‐7973) and anti-p‐JNK (G-7, sc‐6254) from Santa Cruz Biotechnology Inc. (Dallas, USA); anti-LC3B (ab48394) from Abcam (Cambridge Science Park, England) and anti-p-USF1 (12651) from Signalway Antibody (College Park, Maryland, USA).

**2.6 Cell proliferation, clonogenic, invasion, migration, and wound healing assays**

Cell proliferation, clonogenicity, invasion, migration, and wound healing were assayed as described previously(4).

**2.7 Chromatin immunoprecipitation (ChIP) assays**

Cells were cross-linked with 1% formaldehyde and quenched in glycine solution. ChIP assays were performed using the Pierce Magnetic ChIP Kit (Thermo Fisher Scientific, Massachusetts, USA) according to the manufacturer’s protocol. Briefly, cells were cross-linked, lysed and sonicated to obtain DNA samples. Anti-MEF2A (12382-1-AP, Proteintech), Anti-USF1, Anti-acetyl Histone H3 antibody（06-599, Merck-MilliporeMillipore, Hangzhou, China） and normal IgG antibody (MultiSciences) were applied for immunoprecipitation. ChIP-enriched DNA samples were analyzed [respectively](javascript:;) by qRT-PCR to quantify the putative Histone H3-binding in the ATG4B promoter region and USF1 binds to HDAC4 promoter region, and the data were normalized against that of control IgG. Primer sequences for ChIP assays were as follows: ATG4B: forward, 5′-CAAGAAAGTGCGCTGGCTGA-3′; reverse, 5′-AGTTTTAAAGACGGGAATGG-3′. HDAC4: forward, 5′-AAATGGCACGCAGCAATGGG-3′; reverse, 5′-AGTTTTTCACGTACAAAGAG-3′.

**2.8 Luciferase reporter assay**

Luciferase reporter assay was performed as described previously(2). In brief, the 48-well plate was inoculated at a density of 3 × 10^5^ cells/well and transfected with WT-ATG4B or Mut-ATG4B promoter luciferase plasmid and MEF2A plasmid or control vector, then co-transfected with Renilla luciferase. WT-HDAC4 or Mut-HDAC4 promoter luciferase plasmid was transfected to GC cells with USF1 plasmid or control vector, then co-transfected with Renilla luciferase. After transfection for 48 h, the luciferase activity was measured using the Dual-Luciferase Assay Kit (Beyotime).

**2.9 Tissue microarray (TMA) construction and immunohistochemistry (IHC) analysis**

The TMA system from Quick-Ray (UT06; UNITMA, Korea). Specific experimental methods of TMA and IHC were performed according to a previously described protocol(5). Tumor sections of patients, nude mouse xenografts, lung metastasis and peritoneal implantation metastasis were analyzed by IHC using the EnVision™ System (Dako, Carpinteria, CA). All primary antibodies for IHC were shown below: anti-HDAC4 (1:100), anti-LC3B (1:200, ab48394, Abcam), anti-MEKK3 (1:100), anti-ATG4B (1:100) and anti-Ki67 (1:100, ab15580, Abcam). IHC results were evaluated by two independent pathologists using the following criteria: 3 (strong positive), 2 (moderate positive), 1 (weak positive) and 0 (negative). The score was then based on the positive rate: 4 (>75%), 3 (>50%-75%), 2 (>25%-50%), 1 (5%-25%), 0 (<5%). Final score: positive score multiplied by staining intensity score, HDAC4 high expression: score ≥ 3; HDAC4 low expression score ≤ 2(6, 7). At the end of the study, the two pathologists reviewed the discordant cases together under a multiheaded microscope and invited a third pathologist to re-evaluate when major discrepancies appeared.

**2.10 Coimmunoprecipitation (Co-IP) analysis**

Total cells were lysed and obtained supernatant was incubated with the negative control IgG or specific antibodies overnight. Then, protein A+G agarose (Bioworld Technology, St. Louis Park, MN, USA) were added and incubated for 2h at 4℃. The protein-antibody complexes formed were washed in PBS, and the cleaned beads were centrifuged. Finally, the supernatant was removed, and the samples were subjected to sodium dodecyl sulfate-polyacrylamide gel electrophoresis.

**2.11 Autophagic flux analysis**

GFP-mRFP-LC3 lentivirus was purchased from GeneChem. GC cells SGC7901 and BGC823 were inoculated on the cover of a 24-well plate and 48h were treated with HDAC4 koncdown in the presence or absence of 10 nM BAF-A1. After washing with PBS thrice, the cells were fixed with 4% paraformaldehyde for 20 min at room temperature. The nuclei were retrostained with DAPI for 5 min and rinsed with PBS thrice. The autophagosomes (yellow spots) and autolysomes (red spots) were observed under a confocal microscope. The yellow and red spots were counted using ImageJ software.

**2.12 In vivo tumorigenesis and metastasis assays**

Four-week-old, male, athymic, nude mice were purchased from the Animal Center of Medical College of Nantong University and housed under temperature and humidity-controlled conditions. In the tumor formation experiment, 24 nude mice were selected and randomly divided into four groups, the right back of nude mice was transfected with lentivirus containing Sh-HDAC4 or Sh-NC. Tumor volume was measured every three days from day 7. After 22 days of injection, nude mice were humanely killed, and the xenografts were dissected, weighed, and subjected to IHC analysis. In the tumor metastasis experiment, 28 nude mice were selected and randomly divided into four groups. These cells were injected into the nude mice through the caudal vein. After 6 weeks, the mice were humanely killed, and the lungs were subjected to HE staining. Pulmonary nodules were collected and counted under the microscope. Peritoneal implantation metastasis as described previously(6). The Animal Protection Society of Nantong University approved all animal experimental procedures.

**2.13 Transmission electron microscopy (TEM)**

Subcutaneous tumor cells from nude mice were fixed at 4°C with 2.5% glutaraldehyde and postfixed with 1% osmium tetroxide overnight. The samples were dehydrated with ethanol and sequestered in Epon 812. Ultrathin slices were prepared, stained with 2% lead citrate, and visualized under an H-600 transmission electron microscope (Hitachi Scientific Instruments, Mountain View, CA, USA) by a blinded observer. Electron micrographs of at least four representative independent regions were selected for each section to detect subtle changes in cellular structure(8).

**2.14 Microarray analysis and bioinformatics analysis**

Sequences of transcription factors targeting HDAC4 were predicted using the public databases JASPAR 2020 (www.jaspar.genereg.net), PROMO (www.alggen.lsi.upc.edu), and PAZAR (http://www.pazar.info). The expression of HDACs in GC was analyzed using GEPIA (www.gepia2.cancer-pku.cn/), which based on TCGA and GTEX database, Oncomine (www.oncomine.org) database. GSE 79973 (Shao Q et al, 2016) and GSE81948 (Sacconi A et al, 2017) from Gene Expression Omnibus (GEO) database (https://www.ncbi.nlm.nih.gov/geo/). Kaplan-Meier Plotter database (www.kmplot.com) was used to analyze the relationship between the expression levels of the HDAC4 and the prognosis of GC All data were transformed into Log2 (n+1) values. To identify relevant pathways, data from TCGA STAD database and RNA-seq, respectively, GSEA (Gene Set Enrichment Analysis) generated angenerate an ordered list of all related genes based on their correlation with HDAC4 expression. The “h.all.v6.2.entrez.gmt” were selected as reference gene sets(9). The interaction between p62 and MEKK3 proteins was analyzed using STRING (www.string-db.org/) database, and the gene set was sequenced 1,000 times for each analysis. Correlation analysis between USF1 and HDAC4 was performed using TCGA STAD database.

**2.15 RNA sequencing**

Three pairs of SGC7901/Sh-NC and SGC7901/Sh-HDAC4 were prepared for RNA-seq analysis by Genewiz (Suzhou, China). A significant difference in mRNA expression (P-value < 0.05 and |log2 FC| >1) between groups was identified using the fold change cut-off.

**2.16 Statistical analysis**

Data were expressed as mean ± standard deviation (SD). All data were analyzed using SPSS 25.0 (Chicago, IL, USA). All experiments except animal experiments and IHC were performed at least three times. The overall survival rate (OS) and disease-free survival rate (DFS) of GC patients were analyzed and calculated by the Kaplan-Meier and log-rank methods. Cox regression model was used to evaluate the prognostic factors associated with GC. The experimental groups were compared using the t-test with p<0.05 considered to indicate statistical significance.

**REFERENCES**

1. Qu F, Zhu B, Hu Y-L, Mao Q-S, Feng Y. LncRNA HOXA-AS3 promotes gastric cancer progression by regulating miR-29a-3p/LTβR and activating NF-κB signaling. Cancer Cell International. 2021;21(1).

2. Hu YL, Feng Y, Chen YY, Liu JZ, Su Y, Li P, et al. SNHG16/miR-605-3p/TRAF6/NF-kappaB feedback loop regulates hepatocellular carcinoma metastasis. J Cell Mol Med. 2020;24(13):7637-51.

3. Hu YL, Feng Y, Ma P, Wang F, Huang H, Guo YB, et al. HAX-1 promotes the migration and invasion of hepatocellular carcinoma cells through the induction of epithelial-mesenchymal transition via the NF-kappaB pathway. Exp Cell Res. 2019;381(1):66-76.

4. Liu JZ, Hu YL, Feng Y, Jiang Y, Guo YB, Liu YF, et al. BDH2 triggers ROS-induced cell death and autophagy by promoting Nrf2 ubiquitination in gastric cancer. J Exp Clin Cancer Res. 2020;39(1):123.

5. Xian H, Zhang H, Zhu H, Wang X, Tang X, Mao Y, et al. High APRIL expression correlates with unfavourable survival of gastrointestinal stromal tumour. Pathology. 2014;46(7):617-22.

6. Lu H, Feng Y, Hu Y, Guo Y, Liu Y, Mao Q, et al. Spondin 2 promotes the proliferation, migration and invasion of gastric cancer cells. J Cell Mol Med. 2020;24(1):98-113.

7. Zhu LF, Ma P, Hu YL, Feng Y, Li P, Wang H, et al. HCCR-1 is a Novel Prognostic Indicator for Gastric Cancer and Promotes Cell Proliferation. J Cancer. 2019;10(15):3533-42.

8. Liu JZ, Hu YL, Feng Y, Guo YB, Liu YF, Yang JL, et al. Rafoxanide promotes apoptosis and autophagy of gastric cancer cells by suppressing PI3K /Akt/mTOR pathway. Exp Cell Res. 2019;385(2):111691.

9. Subramanian A, Tamayo P, Mootha VK, Mukherjee S, Ebert BL, Gillette MA, Paulovich A, Pomeroy SL, Golub TR, Lander ES, Mesirov JP. Gene set enrichment analysis: a knowledge-based approach for interpreting genome-wide expression profiles. Proc Natl Acad Sci U S A. 2005;10(10).

**Supplementary Figure Legends**

**Supplementary Figure 1: Expression and prognostic value of related molecules of the HDAC family in GC.**

A-Q. Expression of HDACs between GC and normal tissues in GEPIA (N, normal tissue; T, tumor tissue). R and S. Heatmap of differentially expressed HDACs in GC based on microarray datasets (GSE79973 and GSE81948).

**Supplementary Figure 2: Effect of HDAC4 on GC cells proliferation, migration, and invasion.**

A, B. HDAC4 was upregulated in GC cell lines compared with normal gastric epithelial GES-1 cells as shown by qRT-PCR and western blot analyses. C. Western blotting analysis of the relative expression of HDAC4 protein after GC cells were transfected with Sh-HDAC4, Sh-HDAC4#2 and Sh-HDAC4#3 lentivirus. D, E. CCK-8 and colony formation assays for assessing cell proliferation. F. Wound healing assay of the ability of GC cells to migrate (scale bar, 50μm). G. Transwell assay of the migration and invasion ability of GC cells. Scale bar, 50μm. ***p*<0.01, ***p*<0.001.

**Supplementary Figure 3: Quantification of GC cells functional experiment**

A. GSEA of HDAC4 and MAPK pathways according to the TCGA databases. B. Western blot showed that anisomycin can active P38 MAPK. C, D. Quantification of the colony formation abilities. E, F. Quantification of transwell assays shown. G, H. The expression of MEKK1/2/3/4 was dectect by qRT-PCR in GC cells.

**Supplementary Figure 4: The protein level of MEKK3 in different treatment groups was detected by western blot.**

A, B. Western blot analysis of MEKK3 after treatment with DMSO and MG132 before harvest in GC cells; GAPDH was used as control. C. QRT-PCR was used to detect the expression of autophagy-related genes in GC cells with or without HDAC4 knockdown. **P*<0.05, ****P*<0.01, #not significant.
