# Supplementary figures and images for: HDAC4 promotes the growth and metastasis of gastric cancer via autophagic degradation of MEKK3
Source: Br J Cancer. 2022 May 30;127(2):237–48. doi: 10.1038/s41416-022-01805-7 (PMC9296555; doi:10.1038/s41416-022-01805-7)

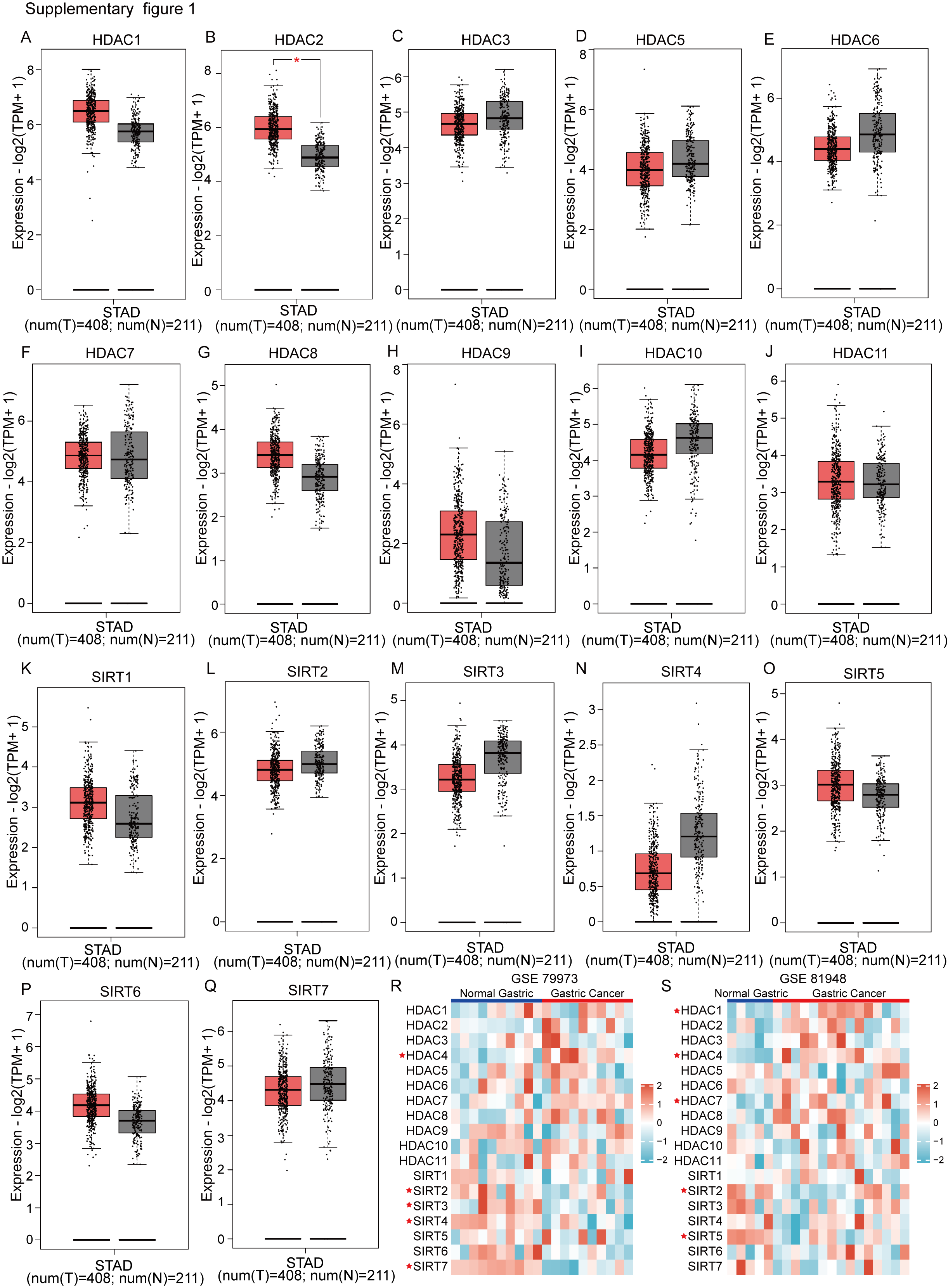

Supplement: Supplementary file 2 — Supplementary figure 1.tif [file 41416_2022_1805_MOESM2_ESM.tif]

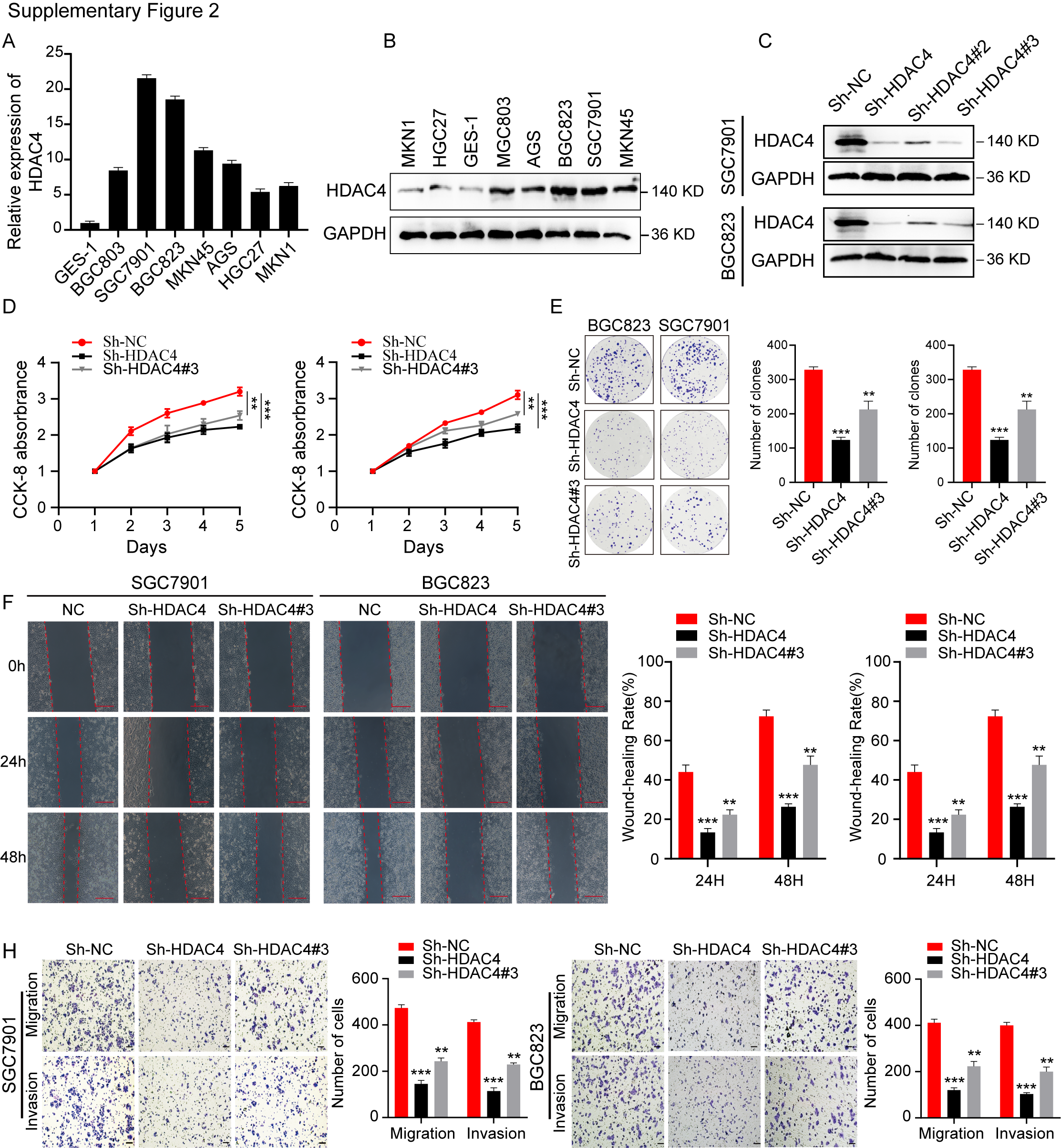

Supplement: Supplementary file 3 — Supplementary figure 2.tif [file 41416_2022_1805_MOESM3_ESM.tif]

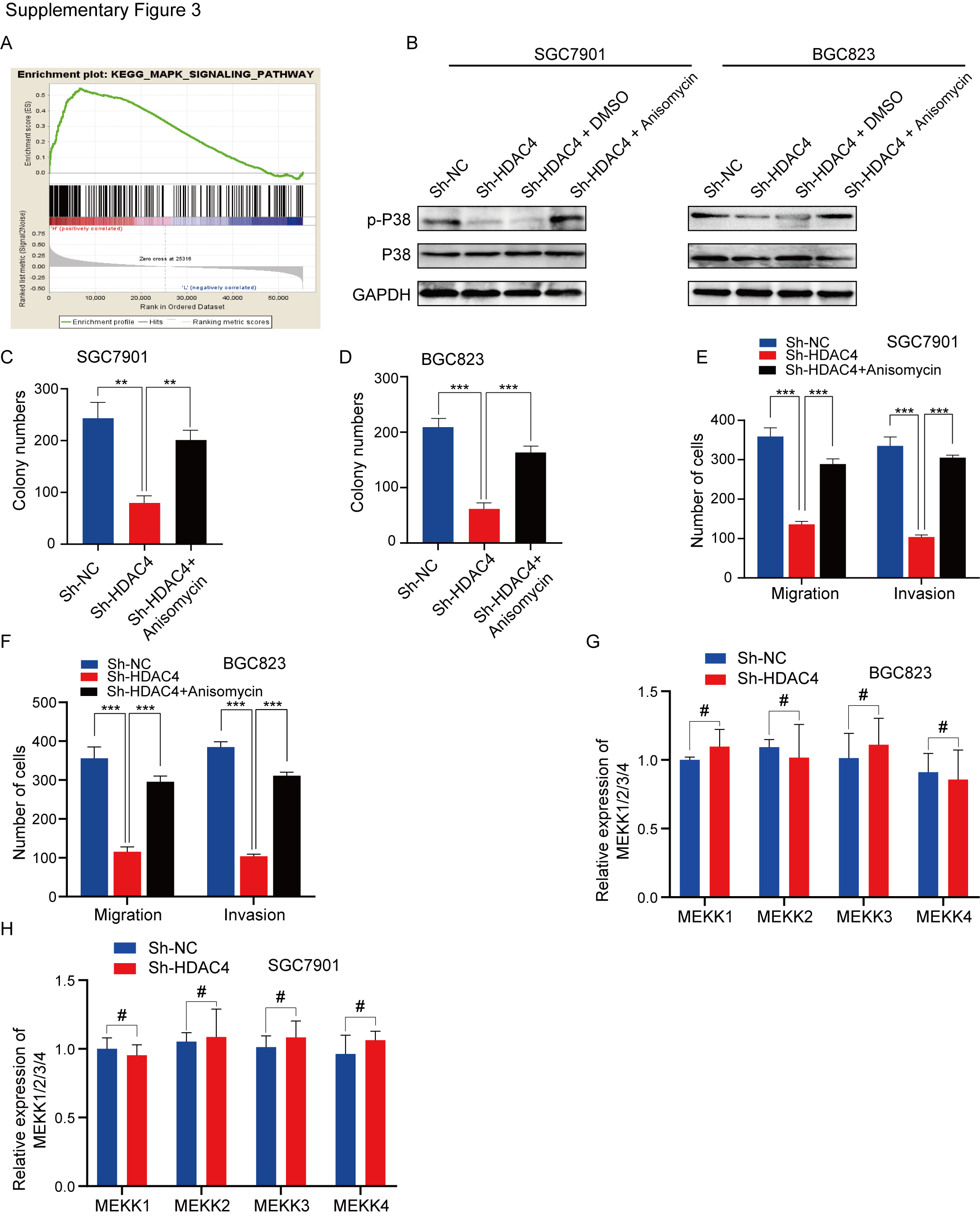

Supplement: Supplementary file 4 — Supplementary figure 3.tif [file 41416_2022_1805_MOESM4_ESM.tif]

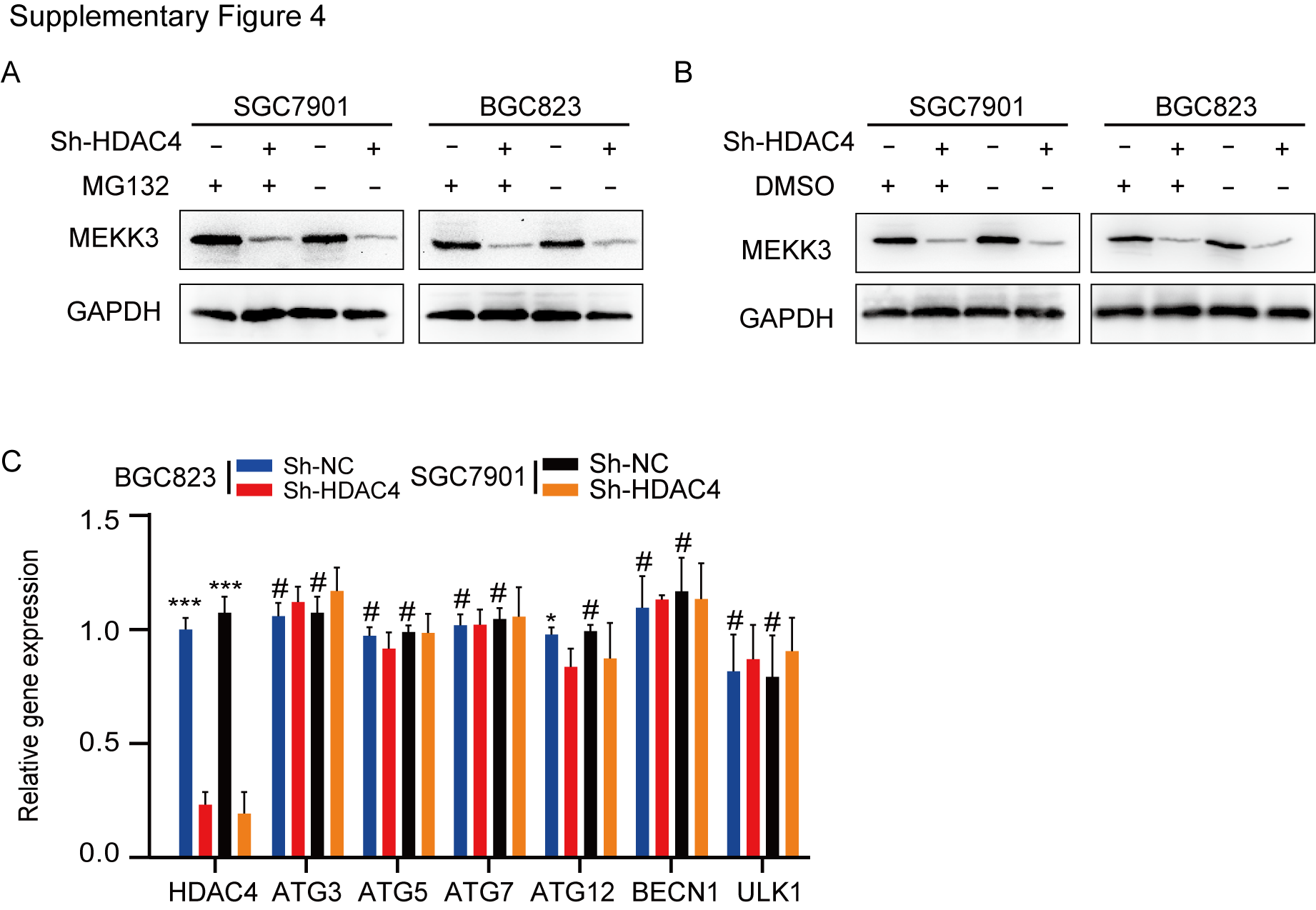

Supplement: Supplementary file 5 — Supplementary figure 4.tif [file 41416_2022_1805_MOESM5_ESM.tif]
